# Infection of *Cronobacter sakazakii* ST1 Producing SHV-12 in a Premature Infant Born from Triplet Pregnancy

**DOI:** 10.3390/microorganisms9091878

**Published:** 2021-09-04

**Authors:** Monika Lachowska, Radosław Izdebski, Paweł Urbanowicz, Dorota Żabicka, Barbara Królak-Olejnik

**Affiliations:** 1 Department of Neonatology, Wrocław Medical University, 50-556 Wrocław, Poland; monika.lachowska@umed.wroc.pl (M.L.); barbara.krolak-olejnik@umed.wroc.pl (B.K.-O.); 2 Department of Molecular Microbiology, National Medicines Institute, 00-725 Warsaw, Poland; p.urbanowicz@nil.gov.pl; 3 Department of Epidemiology and Clinical Microbiology, National Medicines Institute, 00-725 Warsaw, Poland; d.zabicka@nil.gov.pl

**Keywords:** *Cronobacter sakazakii*, neonatal infection, ST1, whole genome sequencing, WGS

## Abstract

*Cronobacter sakazakii* can cause severe life-threatening invasive infections in neonates, with a high mortality rate mostly associated with powdered infant formula consumption. The study describes a fatal *C. sakazakii* infection in premature infant fed only with expressed human milk. Despite the identification of etiological factor from patient’s blood, the epidemiological investigation, including mother’s skin, hospital surfaces, milk expressing devices, and milk samples, did not show bacterial contamination. The infection was caused by *C. sakazakii* ST1, being one of the leading genotypes reported in invasive infections. The phylogenetic analysis of the international collection of the ST1 organisms allowed us to identify the isolate as a member of the main cluster. The pathogenic potential of the isolate was augmented by the presence of IncFIB-like molecule representing virulence plasmids of pESA-3 family. Isolate presented ESBL phenotype associated with *bla*_SHV-12_ gene harboured by IncX3 plasmid. The described case gave valuable information to genetics of Cronobacter, and also urges the need of wider whole-genome sequencing implementation as a part of diagnostic procedure.

## 1. Introduction

*Cronobacter sakazakii*, previously known as *Enterobacter sakazakii*, is a Gram-negative bacillus of the *Enterobacterales* order regarded as an opportunistic neonatal pathogen, affecting mostly infants born prematurely, less often older infants or elderly individuals [1,2,3]. The invasive infection in infants can be manifested as meningitis, necrotizing meningoencephalitis, septicemia, necrotizing entercolitis, cerebral infarctions, or brain abscesses [2]. These may progress rapidly, with death in a few hours from the first signs of infection, and a mortality rate 40–80% [1,3,4,5]. The life-threatening infections caused by *C. sakazakii* are very rare, with less than 200 cases described to date [3,4]. A large number of such reports concerned children consuming powder infant formula (PIF), however, in recent years some isolates have also been identified in neonates fed exclusively with human milk [3,6,7]. The genotypes isolated from PIF, associated with neonatal septicaemia or meningitis cases, had been classified to a few groups of related organisms, of which ST4 and ST1 are the most common [2,8]. Molecular studies of *C. sakazakii* genomes allowed us to define several virulence factors, which may play crucial roles in infections and enhance the pathogenicity of strains [2]. To the most important belong chromosomal genes involved in the biosynthesis of fimbriae, adhesion, and biofilm formation, and those localized on virulence plasmids of the pESA-3 family responsible for the synthesis and secretion of the siderophore, named Cronobactin [2].

## 2. Case Presentation

A thirty-six year-old woman became pregnant after infertility treatment consisting of ovulation stimulation and intrauterine insemination. Pregnancy was complicated by a history of cervical insufficiency (cervical cerclage in 30th week of pregnancy) and cervical infections (*Klebsiella pneumoniae*, *Enterococcus foecium*—both widely susceptible) successfully treated with antibiotics.

At 34 weeks and 3 days, a gestational age premature baby girl was born by caesarean section as a first of the triplets, with birth weight 1720 g and Apgar score of 9, 8, and 9 at 1, 5, and 10 min, respectively. After delivery room stabilization, she was placed into an incubator with passive oxygen therapy. The newborn received total parenteral nutrition and trophic feeding of expressed mother’s colostrum in the first day of life, then was supported with donor human milk from the Human Milk Bank through orogastric feeding tube. On the second day of life, patient received phototherapy due to abnormal total serum bilirubin concentration (10.2 mg/dL). At the end of the third day, the infant’s health suddenly deteriorated, requiring intubation and ventilation. From the first symptoms and abnormal infections markers (high CRP—247.5 mg/L, PCT—17.14 ng/mL, low leukocyte—3.4 G/L, and platelet count—29 g/L) she was treated with meropenem (40 mg/kg/dose twice a day) and vancomycin (15 mg/kg/dose twice a day). Blood culture was positive and *C. sakazakii* NMI5563_17 isolate had been identified on the sixth day of life. Phenotypic detection of ESBL was carried out by the ESBL double-disc synergy test (DDST) as described previously [9]. The antibiotic susceptibility testing was evaluated using gradients tests and broth microdilution method for colistin (http://www.eucast.org/; accessed on 1 June 2021). Isolate showed resistance to ampicillin, piperacillin, ceftazidime, cefotaxime, and aztreonam (Table 1).

On the fifth day of life, the platelet transfusion was performed. The culture of cerebrospinal fluid was negative. Despite appropriate antimicrobial treatment with meropenem (according to the susceptibility profile of the bacteria), the infant had developed sepsis with multiorgan failure and meningitis with multiple brain abscesses. Cerebral ultrasound showed multiple brain abscesses with disintegration tendency and complete obliteration of brain structures. The electroencephalography examination showed low voltage signal. After discussion with her parents, care was redirected to palliation, and the infant died at 12 days of age.

The other triplets (II-female 1500 g and III-male 1430 g) have been fed in the same way (orogastric tube and own mother’s breastmilk supplemented by donor milk) and were in good condition with no symptoms of infection. They were discharged after 27 days of hospitalization.

In the course of epidemiological investigation, mother’s skin (axillary and inguinal regions), hospital surfaces, and equipment were swabbed, milk samples (from mother’s breastmilk and Human Bank Milk) together with milk expressing devices including breast pump kits, underwent microbiological examination. The analysed surfaces, devices, and milk did not show *Cronobacter* contamination.

## 3. Molecular Analysis of the *C. sakazakii* Isolate 

**Figure 1:**
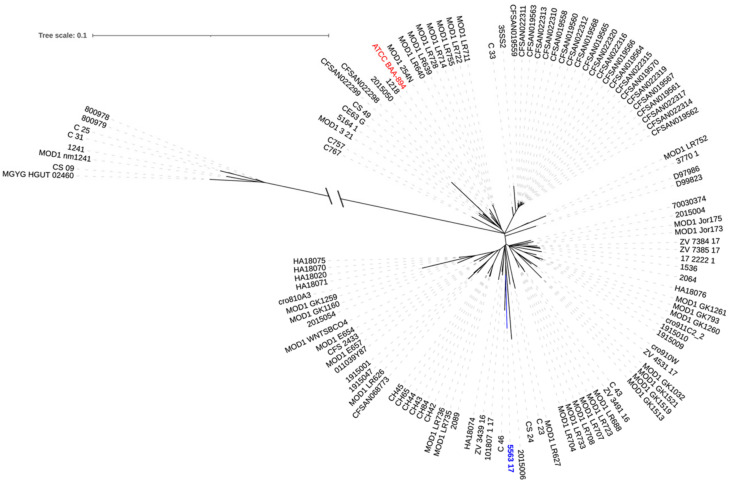
SNP-based phylogenetic tree of *C. sakazakii* ST1 international genomes available in PubMLST and GenBank. The isolate from Poland is marked in blue, the ATCC BAA-894 in red. Numbers on the inner circle correspond to original numbers of the study isolates. The tree was constructed using Parsnp (https://github.com/marbl/harvest-tools) and visualized with iTOL (https://itol.embl.de/).

The isolate NMI5563_17 was sequenced by Illumina MiSeq (Illumina, San Diego, CA, USA) with 50× coverage and MinION (Oxford Nanopore Technologies, Oxford, UK). Hybrid reads were assembled using Unicycler v.0.4.7 (https://github.com/rrwick/Unicycler), resulting circular chromosome of 4,315,383 bp. The MLST analysis classified the isolate to ST1 (https://pubmlst.org/cronobacter/). The core-genome phylogeny was performed using Parsnp v.1.2. (https://github.com/marbl/parsnp) with NMI5563_17 chromosome as a reference and all available at PubMLST (*n* = 111) and GenBank (*n* = 8) *C. sakazakii* ST1 non-duplicates genomes (as of 23 August 2021; Figure 1). The analysis revealed two separate clades: the main one, including the isolate NMI5563_17 and 111 international genomes, and an outlier group, containing eight records. The SNP analysis revealed 243–2480 polymorphic positions between any individual genome and the reference NMI5563_17 within ~4.3 Mb (73%) of the reference sequence (Table S1). The mean SNP value in the main clade was 334, and the median was 333, whereas the same in the second clade were 2428 and 2441, respectively. The closest relatives of Polish isolate were two strains from China, C.46 and 2015,006 distanced by 243 and 381 SNPs, respectively.

The long-read sequencing of NMI5563_17 isolate revealed four complete plasmids assemblies, namely pCS-WR1 (127,980 bp), pCS-WR2 (45,949 bp), pCS-WR3 (43,705 bp), and pCS-WR4 (3760 bp), classified by PlasmidFinder 2.1 (https://cge.cbs.dtu.dk/services/PlasmidFinder/) to IncFIB-like, IncFIB, IncX3, and non-typable incompatibility groups, respectively. GenBank screenings for plasmid sequence comparisons performed by BLASTn revealed pCS-WR1 99.98% identity to virulence plasmid pESA3 (NC_009780). Similar to pESA3, pCS-WR1 harboured putative virulence factors: two iron acquisition systems: *eitCBAD* and *iucABCD/iutA*, plasmidogen activator *cpa* gene locus, and truncated T6SS secretion system (comprised of 13/16 ORFs; Figure S1). The pCS-WR2 contained silver *silCBAP/RS* and copper *cpoABCDRS* resistance operons, similarly to pCTU3 (NC_013285) and pSP291-2 (NC_020261), while pCS-WR3 carried *bla*_SHV-12_ and *qnrS1* loci, as indicated by ABRicate v1.0.1 (https://github.com/tseemann/abricate).

## 4. Discussion

The risk factors, clinical picture, and fatal outcome in the described case was typical for *C. sakazakii* infection in neonates, confirmed by identification of etiological factor from the patient’s blood. The epidemiological investigation was focused on finding the source of infection and way of transmission of the pathogen, but none of the taken samples were positive. The infant was fed with human milk, but expressed breast milk and donor milk, as well as milk expressing devices including breast pump kits, did not show any bacterial contamination. The patient did not receive PIF, thus this transmission vehicle was excluded as well. Despite the efforts made, the source of the infection remains unknown.

The comparative analysis of the isolate with all available genomes of *C. sakazakii* ST1, located NMI5563_17 within the main clade. The isolate formed a separate branch with genomes from China, New Zealand, USA, Austria, and Slovenia, however genetic distances (243-381 SNPs) do not allow us to indicate the exact origin of the Polish strain.

In general, *C. sakazakii* isolates are susceptible to the most commonly clinically used antimicrobial agents, with only few studies so far reporting strains with acquired resistance genes [2]. Our genomic analysis of global *C. sakazakii* ST1 population, revealed 36 (~30%) isolates from ten countries with single acquired gene, mainly *mcr*-9.1 (*n* = 35; Table S1). The majority of MCR-producers (>60%) were collected recently, between 2017 and 2020 [10]. The exception was an isolate from Switzerland with six acquired genes conferring resistance to different antimicrobial compounds. The *bla*_SHV-12_ gene, one of the most predominant ESBL within *Enterobacterales*, has been observed only in studied isolate, being, to our knowledge, the first report in ST1. The presence of the *bla*_SHV-12_ gene on conjugative IncX3 plasmid (pCS-WR3) is a novelty within a *Cronobacter* genus, but not in other *Enterobacterales*. IncX3 plasmids had been broadly identified in this order as potent vectors for AMR genes, including ESBL and/or carbapenemases, being highly stable and costless for the bacterial host [11]. Together with the pCS-WR1 of the previously described pESA-3 family virulence plasmids [2], pCS-WR3 could have a possible impact on the course of infection by increasing pathogenic potential and drug resistance of the isolate.

## Figures and Tables

**Table 1:** Antibiotic susceptibility of *C. sakazakii* NMI5563_17 isolate.

Antibiotic	Minimal Inhibitory Concentration (MIC; mg/L)	Interpretation According to EUCAST Breakpoints
Ampicillin	>256	Resistant
Amoxicillin-clavulanic acid	4	Susceptible
Ampicillin-sulbactam	2	Susceptible
Piperacillin	128	Resistant
Piperacillin-tazobactam	2	Susceptible
Ceftazidime	64	Resistant
Cefotaxime	12	Resistant
Cefepime	1	Susceptible
Aztreonam	64	Resistant
Ertapenem	0.032	Susceptible
Imipenem	0.125	Susceptible
Meropenem	0.032	Susceptible
Amikacin	2	Susceptible
Gentamicin	0.25	Susceptible
Tobramycin	0.5	Susceptible
Ciprofloxacin	0.25	Susceptible
Levofloxacin	0.5	Susceptible
Tetracycline	4	Susceptible ^1^
Tigecycline	0.25	Susceptible
Colistin	0.25	Susceptible
Chloramphenicol	8	Susceptible
Trimetoprim-sulfamethoxazole	0.125	Susceptible

^1^ CLSI breakpoint interpretation (https://clsi.org/; accessed on 1 June 2021).

## Data Availability

Genomic sequences have been deposited in the NCBI under the BioProject and BioSample numbers PRJNA645121 and SAMN15493123, respectively. Sequences of NMI5563_17 isolate plasmids are available under the following GenBank accession numbers: pCS-WR1, MT759836; pCS-WR2, MT759837; pCS-WR3, MT759838; pCS-WR4, and MT759839.

## Supplementary Materials

The following materials are available online at https://www.mdpi.com/article/10.3390/microorganisms9091878/s1, Figure S1: Comparison of the pESA3 plasmid to pCS-WR1 and previously reported similar plasmids from *Cronobacter* species, Table S1: *C. sakazakii* ST1 isolates included in the genomic study with numbers of SNPs, acquired resistance genes and basic epidemiological data.
