# Supplementary material for: Infection of Cronobacter sakazakii ST1 Producing SHV-12 in a Premature Infant Born from Triplet Pregnancy
Source: Microorganisms. 2021 Sep 4;9(9):1878. doi: 10.3390/microorganisms9091878 (PMC8469300; doi:10.3390/microorganisms9091878)
Supplement: Supplementary file 1 [file microorganisms-09-01878-s001.zip › microorganisms-1338153-supplementary.pdf]

microorganisms MDPI

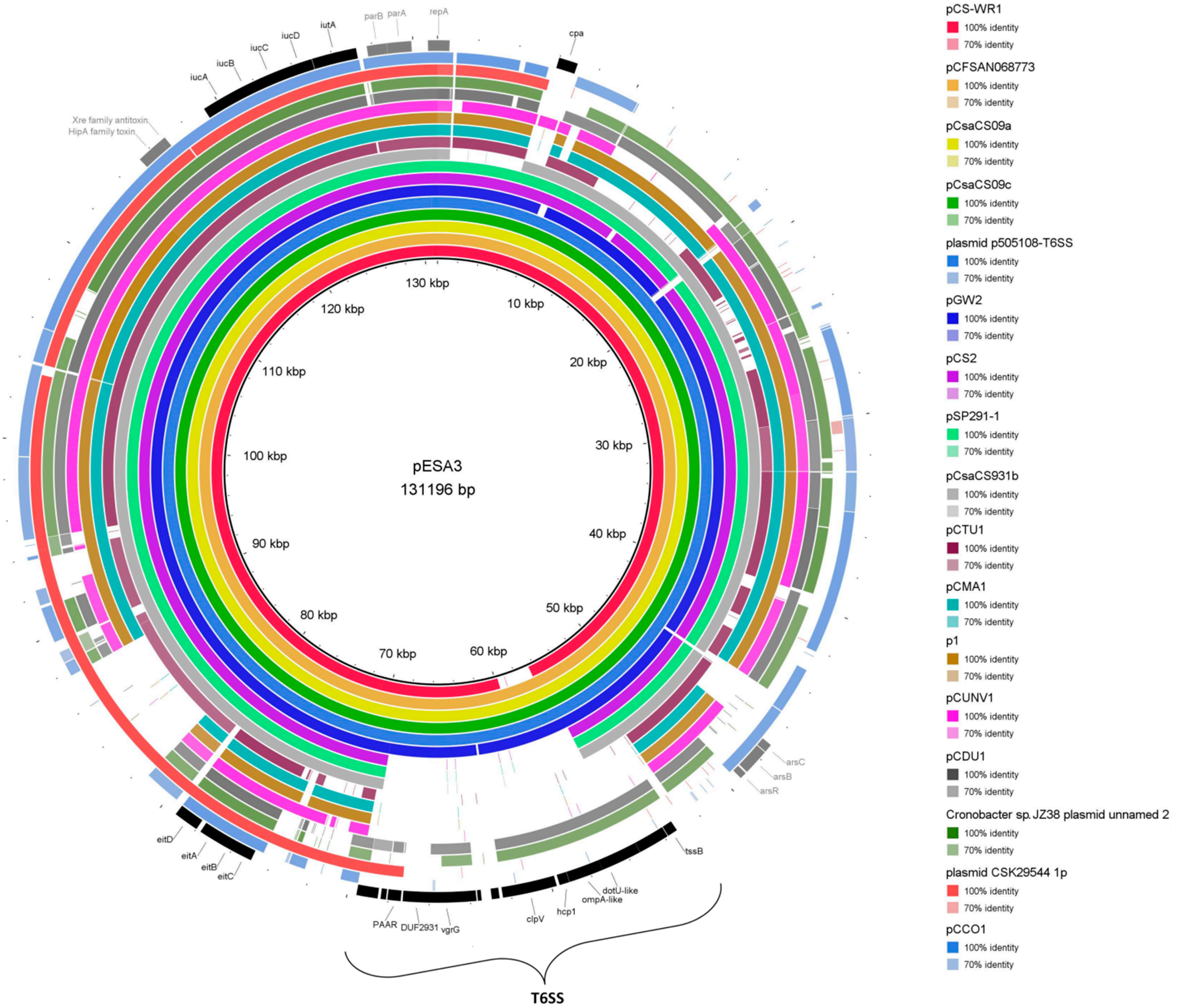

Figure S1. Comparison of the pESA3 plasmid (inner, thin black circle) to pCS-WR1 and previously reported similar plasmids from *Cronobacter* species. The outer thick ring refers to the annotation of pESA3 - black colour represents virulence factors, gray - plasmid maintanance plus *arsCBR* operon. The percentage of sequence identity is reflected by color intensity. The picture was created using BRIG software (http://brig.sourceforge.net/).

Table S1. *C. sakazakii* ST1 isolates included in the genomic study with numbers of SNPs, acquired resistance genes and basic epidemiological data.

Isolate	Number of SNPs	Acquired resistance genes	Country	Source	Year	Database
NMI5563_17	reference	*bla*SHV-12, *qnrS1*	Poland	Clinical	2018	PubMLST
C.46	243	-	China	Food	2006	PubMLST
MOD1_Jor173	288	-	Jordan	Food	-	PubMLST
MOD1_Jor175	289	-	Jordan	Food	2008	GenBank
ZV-3439-16	297	-	Slovenia	Food	2016	PubMLST

MOD1_LR707	298	-	USA	Food	2006	PubMLST
MOD1_LR708	299	-	USA	Food	2006	PubMLST
MOD1_LR723	299	-	USA	Food	2004	PubMLST
MOD1_LR688	300	-	USA	Food	2004	PubMLST
101807-1-17	300	-	Austria	Clinical	2017	PubMLST
70030374	300	-	Norway	Clinical	2011	PubMLST
MOD1_LR704	301	-	USA	Food	2004	GenBank
MOD1_LR733	303	-	USA	Food	2007	PubMLST
2015004	304	*mcr*-9.1	China	Food	2020	PubMLST
011039Y87	308	*mcr*-9.1	Denmark	Clinical	1987	PubMLST
CFS-2433	308	*mcr*-9.1	Canada	Clinical	2009	PubMLST
MOD1-E657	308	*mcr*-9.1	Ireland	Clinical	2019	PubMLST
MOD1-E654	309	*mcr*-9.1	Ireland	Clinical	-	PubMLST
ZV-7384-17	312	-	Slovenia	Food	2017	PubMLST
ZV-7385-17	312	-	Slovenia	Food	2017	PubMLST
C.43	313	*mcr*-9.1	China	Food	2006	PubMLST
CH44	313	*mcr*-9.1	Chile	-	2017	PubMLST
CH45	313	*mcr*-9.1	Chile	-	2017	PubMLST
CH65	313	*mcr*-9.1	Singapore	-	2017	PubMLST
CS-24	314	-	New Zealand	-	2005	PubMLST
CH42	314	*mcr*-9.1	Chile	-	2017	PubMLST
CH43	314	*mcr*-9.1	Chile	-	2017	PubMLST
CH84	315	*mcr*-9.1	Chile	-	2017	PubMLST
2015050	317	-	China	Food	2020	PubMLST
HA18074	318	*mcr*-9.1	China	Food	2020	PubMLST
MOD1_LR626	318	-	USA	Food	2004	GenBank
1536	319	-	Germany	Environmental	2009	PubMLST
MOD1_WNTSBCO4	319	*mcr*-9.1	USA	Food	2015	PubMLST
MOD1_LR736	319	*mcr*-9.1	USA	Food	2018	PubMLST
ZV-4531-17	320	-	Slovenia	Food	2017	PubMLST
17-2222-1	320	bla_{TEM-1}, *dfrA16*, *ere(A)*, *qnrS1*, *sul1*, *tet(D)*	Switzerland	Food	2017	PubMLST
2089	321	*mcr*-9.1	France	Clinical	2004	PubMLST
ZV-3491-16	321	*mcr*-9.1	Slovenia	Food	2016	PubMLST
MOD1_LR735	322	*mcr*-9.1	USA	Food	2004	PubMLST
HA18076	323	-	China	Food	2020	PubMLST
1915009	323	*mcr*-9.1	China	Food	2019	PubMLST
1915010	323	*mcr*-9.1	China	Food	2019	PubMLST
MOD1_LR640	324	-	USA	Food	2005	PubMLST
MOD1_LR639	325	-	USA	Food	2005	PubMLST
MOD1_GK1259	325	*mcr*-9.1	Germany	Environmental	2014	PubMLST
MOD1_LR752	326	-	USA	Food	2008	PubMLST

MOD1_LR755	326	-	USA	Food	2004	PubMLST
C.23	326	-	China	Food	2006	PubMLST
MOD1_GK1160	326	*mcr*-9.1	Germany	Environmental	2014	PubMLST
2064	327	-	France	Environmental	-	PubMLST
MOD1_LR711	327	-	USA	Food	2004	GenBank
ATCC BAA-894	328	-	USA	Food	2001	PubMLST
1218	328	-	USA	Clinical	2001	PubMLST
MOD1_LR722	328	-	USA	Food	2006	PubMLST
MOD1_LR714	328	-	USA	Food	2004	GenBank
CFSAN068773	331	-	USA	-	1996	PubMLST
cro911C2-2	333	*mcr*-9.1	China	Food	2012	PubMLST
cro910W	335	-	China	Food	2012	PubMLST
C.33	335	-	China	Food	2006	PubMLST
1915001	336	*mcr*-9.1	China	Food	2019	PubMLST
MOD1-254N	337	-	Ireland	Clinical	-	PubMLST
CE63-G	337	-	China	Food	-	PubMLST
MOD1_GK793	338	*mcr*-9.1	Germany	Environmental	2019	PubMLST
D97986	339	-	Denmark	Clinical	2017	PubMLST
D99823	339	-	Denmark	Clinical	2017	PubMLST
MOD1_GK1260	339	*mcr*-9.1	Germany	Environmental	2019	PubMLST
MOD1_GK1261	340	*mcr*-9.1	Germany	Environmental	2019	PubMLST
CS-49	341	-	New Zealand	Food	2006	PubMLST
MOD1_3-21	342	-	South Korea	Food	2011	PubMLST
MOD1_LR728	342	-	USA	Food	2004	GenBank
CFSAN019570	344	-	USA	Environmental	2014	PubMLST
CFSAN019558	344	-	USA	Environmental	2014	PubMLST
CFSAN022313	344	-	USA	Environmental	2014	PubMLST
CFSAN019560	344	-	USA	Environmental	2014	PubMLST
MOD1_LR627	345	-	USA	Food	2004	GenBank
CFSAN019561	346	-	USA	Environmental	2014	PubMLST
CFSAN022311	346	-	USA	Environmental	2014	PubMLST
CFSAN022312	346	-	USA	Environmental	2014	PubMLST
CFSAN022320	347	-	USA	Environmental	2014	PubMLST
CFSAN019566	347	-	USA	Environmental	2014	PubMLST
CFSAN022310	347	-	USA	Environmental	2014	PubMLST
CFSAN022319	348	-	USA	Environmental	2014	PubMLST
CFSAN019559	348	-	USA	Environmental	2014	PubMLST
CFSAN019563	348	-	USA	Environmental	2014	PubMLST
5164-1	348	-	Switzerland	Food	2017	PubMLST
CFSAN022315	349	-	USA	Environmental	2014	PubMLST
CFSAN019564	349	-	USA	Environmental	2014	PubMLST
CFSAN022317	349	-	USA	Environmental	2014	PubMLST

CFSAN022316	353	-	USA	Environmental	2014	PubMLST
CFSAN019565	354	-	USA	Environmental	2014	PubMLST
CFSAN019568	354	-	USA	Environmental	2014	PubMLST
CFSAN019562	354	-	USA	Environmental	2014	PubMLST
CFSAN019567	355	-	USA	Environmental	2014	PubMLST
CFSAN022314	355	-	USA	Environmental	2014	PubMLST
HA18020	355	*mcr*-9.1	China	Food	2018	PubMLST
HA18071	355	*mcr*-9.1	China	Food	2018	PubMLST
HA18070	355	*mcr*-9.1	China	Food	2020	PubMLST
HA18075	355	*mcr*-9.1	China	Food	2018	PubMLST
1915047	358	*mcr*-9.1	China	Food	2019	PubMLST
C757	358	-	Mexico	Clinical	2006	PubMLST
C767	359	-	Mexico	Clinical	2006	PubMLST
CFSAN022298	364	-	USA	Environmental	2014	PubMLST
MOD1_GK1519	364	-	Germany	Environmental	2015	PubMLST
MOD1_GK1513	364	*mcr*-9.1	Germany	Envronmental	2015	PubMLST
MOD1_GK1032	365	*mcr*-9.1	Germany	Environmental	2019	PubMLST
MOD1_GK1521	365	-	Germany	Environmental	2015	PubMLST
2015054	366	*mcr*-9.1	China	Food	2020	PubMLST
3770-1	379	-	Switzerland	-	2017	PubMLST
2015006	381	-	China	Food	2020	PubMLST
35SS2	393	-	Switzerland	Environmental	2012	PubMLST
CFSAN022299	460	-	USA	Environmental	2014	PubMLST
cro810A3	472	-	China	Food	2012	PubMLST
800979	2362	-	Austria	Clinical	2009	PubMLST
800978	2364	-	Austria	Clinical	2009	PubMLST
MOD1-nm1241	2393	-	USA	Clinical	2008	GenBank
1241	2439	*ant(6)-Ia*	USA	Clinical	2009	PubMLST
C.25	2443	-	China	Food	2006	PubMLST
C.31	2467	-	China	Food	2006	PubMLST
MGYG-HGUT-02460	2480	-	UK	Clincal	2019	PubMLST
CS-09	2480	-	Mexico	Clinical	2016	PubMLST
